# Effectiveness of Repeated Gonorrhea Cultures in the Third Trimester

**DOI:** 10.1155/S1064744993000195

**Published:** 1993

**Authors:** Juan G. Torres, T. Fleming Mattox, Joseph G. Pastorek II

**Affiliations:** 1 Department of Obstetrics and Gynecology Louisiana State University Medical Center New Orleans LA USA; 2 Section of Infectious Disease Department of Obstetrics and Gynecology Louisiana State University Medical Center 1542 Tulane Avenue New Orleans LA 70112-2822 USA

## Abstract

*Objective:* With the high cost of health care today, the universal prophylactic measures recommended, and the availability of effective treatment should infection occur, the practice of routinely repeating the endocervical gonorrhea (GC) culture in the third trimester of pregnancy may be unwarranted.

*Methods:* To test this hypothesis, we reviewed charts from patients who had received routine prenatal care during a 2-year period at the Lafayette and Opelousas parish health units. Those charts, which had documented results of both the initial and repeat GC cultures, were then used for retrospective review. The results ofthe initial GC culture were compared with that taken in the third trimester. Other data recorded included age, gravidity, race, and history of gonorrhea, syphilis, or multiple sexual partners.

*Results:* Two hundred fifty charts were available for extraction; 130 of these had documentation of both GC cultures. Of the 130 cultures obtained during the initial prenatal visit, only 6 (4.6%) were positive. Of the repeat cultures taken during the third trimester, none were positive. Thirteen patients (10.0%) had a documented history of GC infection; none of them had positive cultures during the study period.

*Conclusions:* Screening for GC during pregnancy is important and appropriate. This is commonly accomplished by taking a GC culture during the initial prenatal visit. Based upon the present study, we found that repeating this culture in the third trimester, even in a relatively high-risk population, seems unnecessary, whether the initial culture is negative or not.


Infectious Diseases in Obstetrics and Gynecology 1:82–84 (1993)
© 1993 Wiley-Liss, Inc.

### Effectiveness of Repeated Gonorrhea Cultures in the Third Trimester

**Juan G. Torres, T. Fleming Mattox, and Joseph G. Pastorek II**

*Department of Obstetrics and Gynecology, Louisiana State University Medical Center, New Orleans, LA*

### Abstract

***Objective:*** **With the high cost of health care today, the universal prophylactic measures recommended, and the availability of effective treatment should infection occur, the practice of routinely repeating the endocervical gonorrhea (GC) culture in the third trimester of pregnancy may be unwarranted.**

***Methods:*** **To test this hypothesis, we reviewed charts from patients who had received routine prenatal care during a 2-year period at the Lafayette and Opelousas parish health units. Those charts, which had documented results of both the initial and repeat GC cultures, were then used for retrospective review. The results of the initial GC culture were compared with that taken in the third trimester. Other data recorded included age, gravidity, race, and history of gonorrhea, syphilis, or multiple sexual partners.**

***Results:*** **Two hundred fifty charts were available for extraction; 130 of these had documentation of both GC cultures. Of the 130 cultures obtained during the initial prenatal visit, only 6 (4.6%) were positive. Of the repeat cultures taken during the third trimester, none were positive. Thirteen patients (10.0%) had a documented history of GC infection; none of them had positive cultures during the study period.**

***Conclusions:*** **Screening for GC during pregnancy is important and appropriate. This is commonly accomplished by taking a GC culture during the initial prenatal visit. Based upon the present study, we found that repeating this culture in the third trimester, even in a relatively high-risk population, seems unnecessary, whether the initial culture is negative or not.** © 1993 Wiley-Liss, Inc.

Key words

Prenatal care, STD screening, *Neisseria gonorrhoeae,* cervical cultures, maternal infection

The prevalence of gonorrhea (GC) during pregnancy varies from 1.0 to 7.0%,[1–3] depending on the patient population studied. Higher rates are found among populations with risk factors such as single status, adolescence, multiple sexual partners, substance abuse, lack of prenatal care, and the presence of other sexually transmitted diseases.[2] Disseminated gonococcal infection has been noted to be more frequent during pregnancy, with up to 40% of the cases occurring in pregnant women.[4] A pregnant woman with gonococcal infection is more likely to experience preterm delivery, premature rupture of membranes, chorioamnionitis, and postpartum infection.[1,3] Therefore, a cervical screening culture is recommended at the time of the first prenatal visit. A repeat culture in the third trimester (after 28 weeks) is also recommended by the Centers for Disease Control (CDC) in high-risk populations.[5] Though the first recommendation of taking an initial culture is almost universally followed, the latter one is not. This study questions the validity of repeating the GC culture in the third trimester.

Address correspondence/reprint requests to Dr. Joseph G. Pastorek II, Section of Infectious Disease, Department of Obstetrics and Gynecology, Louisiana State University Medical Center, 1542 Tulane Avenue, New Orleans, LA 70112-2822.

Received February 15, 1993
Accepted May 17, 1993

### MATERIALS AND METHODS

Two hundred fifty medical charts from patients receiving routine prenatal care from the Lafayette and Opelousas parish health units were obtained for review during the years 1989 and 1990. The charts included those available for review by two of the investigators (J.G.T., T.F.M.) when they were on site at the respective parish health units. As such, the charts represented a relatively, but unstringently, randomized sample of patients attending these clinics.

Information recorded included age, gravidity, parity, race, gestational ages at the time of the initial and repeat culture, and results of these cultures. Also recorded were the presence of risk factors such as a history of GC, syphilis, and multiple sexual partners. Patient charts were automatically excluded if the result of a third-trimester repeat culture was not recorded. Most of these excluded cases involved patients who began their initial prenatal care at the health units but then switched to a private medical office once they obtained their medical assistance certification.

### RESULTS

One hundred thirty charts met the criteria for inclusion in the study. Forty-three patients (33%) presented for their initial culture during the first trimester (≤ 12 weeks). Another 74 patients (57%) presented during the second trimester (13–26 weeks), and 13 patients (10%) had their initial culture taken in the third trimester (27–40 weeks). Eighty-five of the study patients (65.4%) were black, 37 patients (28.5%) were Caucasian, 2 patients (1.5%) were Hispanic, and 6 patients (4.6%) were classified as other races (Asian, Indian, Middle Eastern). One hundred eleven patients (86%) had had three or fewer pregnancies, 47% of which were primigravidas. Ninety-six patients (74%) were between the ages of 15 and 25.

Of the 130 initial cultures taken, 6 (4.6%) were positive. From the 130 repeat cultures taken in the third trimester, none were positive. Since the precise number of patients seen during this time is not recorded, no absolute prevalence of GC in this population could be calculated.

Thirteen patients (10%) had a history of GC infection, 7 patients (5.4%) had a history of syphilitic infection, and 18 patients (13.8%) reported having had multiple sexual partners in the past. All of these patients had negative initial and repeat cultures. Forty-three patients did not indicate whether they had multiple sexual partners; three of them had a positive initial GC culture. Five of the six patients with positive initial cultures had normal, spontaneous vaginal deliveries at term. All of their babies had uncomplicated courses in the nursery. The delivery records of the sixth patient with a positive initial culture could not be located for review.

Though it was not a primary aim of the study, information was retroactively sought concerning the incidence of gonococcal ophthalmitis among neonates in this population. According to interviews with the attending pediatricians in these health units, no case of gonococcal eye infection was noted during the study period. Indeed, neonatal gonococcal infection is historically extremely rare in this population.

### DISCUSSION

Screening for GC during pregnancy is important and appropriate, especially since the majority of female patients with gonococcal colonization are asymptomatic.[3] Screening is best done by obtaining a GC culture during the initial prenatal visit, considering the usual low cost of a Thayer-Martin plate and the standard recovery techniques in use today. Still, the cost of a GC culture is not negligible. If it is to be repeated, there should be compelling rationale.

The incidence of GC in this study is 4.6%, which is consistent with the reported incidence of between 1 and 7.5%.[1–3] Prenatal screening for GC is important because it helps prevent many serious maternal and neonatal gonococcal complications, e.g., preterm delivery, premature rupture of the membranes, chorioamnionitis, and intrauterine growth retardation.[1,3] It has also been recommended that a repeat GC culture be obtained in the third trimester of pregnancy, especially among those patients considered high risk.[1,3] This report indicates that a repeat GC culture in the third trimester is unnecessary, at least in this population of "high-risk" public assistance obstetric patients. Of the 130 patients in the study, none had a positive repeat culture. Even the presence of risk factors, such as history of GC, syphilis, or multiple sexual partners, did not increase the likelihood that the repeat culture would be positive. Though the size of this study population is small, the practice of repeating the GC culture in the third trimester appears to be in need of reevaluation, particularly during these times of budgetary constraints.

Screening for GC during pregnancy is an accepted part of prenatal care in this country. However, how necessary is it to repeat the GC culture in the third trimester? Are the additional time, effort, and expense worth the benefits derived from repeating the culture? This study suggests that repeating the GC culture in the third trimester is not useful as an additional precaution as surveillance for GC during pregnancy. No additional carriers of GC were found in 130 repeat cultures, even though the patient population was primarily of low socioeconomic standing and adolescent, and had other risk factors such as a history of gonorrhea, syphilis, or multiple sexual partners. During these times of limited health care budgets, for indigent care in particular, tests or procedures that are not cost effective should be discontinued. The routine (repeat) performance of the GC culture in the third trimester may be such a test.

### REFERENCES

1. Wendel GD, Cunningham FG: Sexually transmitted diseases in pregnancy. Williams Obstetrics, Suppl 13, 1991.
2. Edwards LE, Barraha MI, Hamann AA, et al.: Gonorrhea in pregnancy. Am J Obstet Gynecol 132:637, 1978.
3. McNeeley SG: Gonococcal infections in women. Obstet Gynecol Clin North Am 16:467–478, 1989.
4. Al-Suleiman SA, Grimes EM, Jonas HS: Disseminated gonococcal infections. Obstet Gynecol 61:48, 1983.
5. Centers for Disease Control: 1989 Sexually transmitted diseases treatment guidelines. MMWR 38(Suppl 8): 1–43, 1989.
